# A PCR-Based Assay Targeting the Major Capsid Protein Gene of a Dinorna-Like ssRNA Virus That Infects Coral Photosymbionts

**DOI:** 10.3389/fmicb.2017.01665

**Published:** 2017-09-01

**Authors:** Jose Montalvo-Proaño, Patrick Buerger, Karen D. Weynberg, Madeleine J. H. van Oppen

**Affiliations:** ^1^Australian Institute of Marine Science Townsville, QLD, Australia; ^2^AIMS@JCU, James Cook University Townsville, QLD, Australia; ^3^College of Science and Engineering, Department of Marine Biology and Aquaculture, James Cook University Townsville, QLD, Australia; ^4^School of BioSciences, University of Melbourne Parkville, VIC, Australia

**Keywords:** *Symbiodinium*, *Heterocapsa circularisquama* RNA virus (HcRNAV), dinorna-like virus, coral, bleaching

## Abstract

The coral-*Symbiodinium* association is a critical component of coral reefs as it is the main primary producer and builds the reef's 3-dimensional structure. A breakdown of this endosymbiosis causes a loss of the dinoflagellate photosymbiont, *Symbiodinium*, and/or its photosynthetic pigments from the coral tissues (i.e., coral bleaching), and can lead to coral mortality. Coral bleaching has mostly been attributed to environmental stressors, and in some cases to bacterial infection. Viral lysis of *Symbiodinium* has been proposed as another possible cause of some instances of coral bleaching, but this hypothesis has not yet been experimentally confirmed. In this study, we used coral virome data to develop a novel PCR-based assay for examining the presence and diversity of a single-stranded RNA (ssRNA) virus by targeting its major capsid protein (MCP) gene. Illumina sequence analysis of amplicons obtained with novel primers showed 99.8% of the reads had the closest taxonomic affinity with the MCP gene of the virus, *Heterocapsa circularisquama* RNA virus (HcRNAV) known to infect dinoflagellates, indicating that dinorna-like viruses are commonly associated with corals on the Great Barrier Reef. A phylogenetic analysis of MCP gene sequences revealed strong coral species specificity of viral operational taxon units (OTUs). This assay allows a relatively easy and rapid evaluation of the presence and diversity of this particular viral group and will assist in enhancing our understanding of the role of viral lysis in coral bleaching.

## Introduction

A balanced microbiome is essential for the health and functioning of corals (Rohwer et al., 2002; Thompson et al., 2014; Blackall et al., 2015). Corals associate with a wide diversity of microbial organisms, including dinoflagellate photosymbionts (*Symbiodinium* spp.), prokaryotes, fungi and viruses. Of these, *Symbiodinium* spp. form an obligate symbiosis with the coral host and provide up to 95% of its nutritional requirements (Pearse and Muscatine, 1971; Muscatine, 1990). A breakdown of the coral-*Symbiodinium* symbiosis (i.e., coral bleaching) is primarily triggered by environmental stressors, such as increased seawater temperature, high light, and low salinity (Glynn, 1996; Brown, 1997; Douglas, 2003; Hoegh-Guldberg et al., 2007). High temperatures damage the *Symbiodinium* photosystem II machinery, leading to an increased production of reactive oxygen species (ROS) that leak into the coral host cell causing oxidative stress (Lesser, 1997). Simultaneous ROS production has been also found in the coral host mitochondria (Lesser, 1997; Downs et al., 2002; Weis, 2008). Additionally, bleaching can sometimes be caused by bacterial infection of the coral rather than environmental stressors (Kushmaro et al., 1996). Further, it has been speculated that viral lysis of *Symbiodinium* may be responsible for some instances of bleaching. The latter hypothesis stems from transmission electron microscopy (TEM) observations of virus-like particles (VLPs) in different tissue layers of healthy and bleached corals (Wilson et al., 2004; Patten et al., 2008; Bettarel et al., 2012; Leruste et al., 2012; Nguyen-Kim et al., 2014; Pollock et al., 2014; Correa et al., 2016). VLP abundance has been seen to increase under acute stressors, such as elevated temperature (Davy et al., 2006) or to play an important role on the effect of ultraviolet radiation on marine virus-phytoplankton interactions (Jacquet and Bratbak, 2003). Likewise, abundance of VLPs increased in freshly isolated *Symbiodinium* under similar stressors (Wilson et al., 2001; Davy et al., 2006; Lohr et al., 2007; Lawrence et al., 2014). Consistent with these observations, metagenomic studies have revealed an increased abundance of viral sequences in metagenomes obtained from heat stressed corals (Vega Thurber et al., 2008, 2009; Littman et al., 2011) and *Symbiodinium* (Correa et al., 2013; Levin et al., 2016).

Among the viral groups that infect *Symbiodinium* is a small (~30 nm diameter) icosahedral single-stranded ssRNA virus related to *Heterocapsa circularisquama*, HcRNAV (Family: *Alvernaviridae*; genus: dinornavirus) (Nagasaki et al., 2004, 2006; Tomaru et al., 2009; Correa et al., 2013; Weynberg et al., 2014; Levin et al., 2016) and the cricket paralysis virus (Levin et al., 2016). These observations provide rationale for testing the possible role of dinorna-like viruses in coral bleaching.

The aim of this study was to develop a PCR-based assay for examining the presence and diversity of the dinorna-like virus targeting *Symbiodinium* by amplicon sequence analysis on the Illumina platform. Viruses lack a universally conserved gene, such as the 16S and 18S ribosomal RNA genes (Edwards and Rohwer, 2005), but some genes are shared among certain viral groups and can be amplified using PCR primers that target conserved regions. Such signature genes have been used to study environmental viral ecology and diversity (Chen et al., 1996; Larsen et al., 2008; Adriaenssens and Cowan, 2014), and include those encoding structural proteins (e.g., portal protein, major capsid protein –MCP, tail sheath protein –TSP), auxiliary metabolism genes (e.g., *phoH, psbA, psbB*) and polymerase genes. For example, some authors have used algal virus-specific PCR primers to amplify the DNA polymerase gene (*pol*) in water samples (Chen and Suttle, 1995; Chen et al., 1996). Similarly, the MCP gene has been used as a marker for assessing phylogenetic diversity in the *Phycodnaviridae* (Larsen et al., 2008), for example *Emiliana huxley*i viruses (Schroeder et al., 2002; Rowe et al., 2011). Here, we targeted the MCP gene of dinorna-like virus by interrogating virome data from three Great Barrier Reef (GBR) coral species for MCP reads that matched HcRNAV, and used these data to design PCR primers. We tested primers on samples from six coral families and provide preliminary insights into patterns of diversity of dinorna-like virus partial MCP gene sequences.

## Materials and methods

### Sample collection

Eight *Porites lutea* (Poritidae) colonies were collected at Davies Reef (February 2015, 4 m depth, 4 × 4 cm^2^ cores from each colony). In addition, individual colonies of *Acropora tenuis* (Acroporidae)*, Acropora hyacinthus* (Acroporidae), *Acropora millepora* (Acroporidae)*, Fungia fungites* (Fungiidae)*, Galaxea fascicularis* (Oculinidae)*, Goniastrea aspera* (Faviidae)*, Pocillopora damicornis* (Pocilloporidae), and *Porites cylindrica* (Poritidae) were collected from Orpheus Island (August 2014, ~5 m depth). Corals from 2014 were air-blasted and snap frozen in liquid nitrogen (LN2) in the field immediately after collection. Corals from 2015 were transported to the National Sea Simulator at AIMS, and placed in flow-through aquaria with artificial lighting and a constant water temperature of 28°C; tissues from these colonies were air-blasted ~3 days after arrival in the aquarium facility. Colonies were considered healthy (i.e., normal pigmentation, no signs of disease) at the time of collection and tissue processing. Viromes were isolated from coral tissue by mechanical disruption of coral tissue, a series of caesium chloride gradient separations and filtration steps for virome isolation (Weynberg et al., 2014). Viral RNA was extracted with the QIAamp viral RNA kit (Qiagen, cat. 52904) and a final DNase step to remove DNA contamination (Ambion, cat. AM1907). Amplification of total RNA genomes was performed using a cDNA synthesis step as described in the Manual of Aquatic Viral Ecology (MAVE) (Culley et al., 2010) and a Random Priming-mediated Sequence-Independent Single-Primer (RP-SISPA) (Weynberg et al., 2014).

### Primer design

Sequences that matched (i.e., ~60% amino acid (aa) identity cut-off) the MCP of HcRNAV (YP_386496.1 NCBI) were extracted from the RNA metaviromes of *A. tenuis* (Weynberg et al., 2014)*, F. fungites* and *G. fascicularis* (SAMN02709832, SAMN04274763, and SAMN04277306) with a BLASTx NCBI viral RefSeq database, and aligned with Sequencher software and MEGA7 (Kumar et al., 2016) to identify conserved regions. Primers were designed in Primer3Plus (i.e., by generating a consensus sequence from the aligned MCP reads as a template for the primer design). Settings were modified to amplify a ~500 bp product. Primer sets were selected after assessing their stability (i.e., GC/AT ratio, melting temperature) to avoid non-specific duplex formations (Rychlik, 1995). Primer binding specificity was checked in a BLASTn search against the nr database at NCBI. Two primer pairs [First: HcUniv-01F (TCCTTGTWTRYWKGATGCKTTTCA) + HcUniv-01R (MGCCAARTCASWCATATTAAAWGGCA); second: HcUniv-02F (YTKCCTCGASCTRYTGGWCC) + HcUniv-01R (MGCCAARTCASWCATATTAAAWGGCA)] were selected after an initial PCR optimization with Orpheus Island SISPA-amplified templates, as these yielded an amplicon size of ~500 bp (see below).

### PCR optimization

The Qiagen Multiplex Kit was used to generate a ~500 bp amplicon by determining the best performing cycling temperatures and cDNA concentrations; annealing temperature was obtained with a PCR run using a gradient of temperatures (60-58-56-54°C) and primer dilutions (2-4-6-8-10 μM). The best performing cycling condition was enhanced by the use of a nested PCR using the following two primer pairs:

1. First round: HcUniv-01F + HcUniv-01R: 95°C 15 min, [94°C 30 s, 60°C 90 s, 72°C 90 s] 30 cycles, 72°C 10 min and 25°C-end; 10 μM of each primer.
2. Second round: HcUniv-02F + HcUniv-01R; using 1st round profile with 25 cycles only and the same primer dilution. These primers include the corresponding Illumina adaptors for NGS.

The drawback to the nested PCR is that the bias due to preferential amplification may be greater when two successive PCR reactions are applied (Fan et al., 2009). However, to date the potential bias of nested PCR combined with next generation sequencing technologies on the interpretation of viral diversity and structure has not been rigorously examined. There is the possibility, therefore, of over-amplifying certain OTUs. PCR products were run against a 100 bp Plus DNA Ladder in a 1% TBE-agarose gel, 90 V for 40 min, to assess amplicon size and quality. Sequencing was carried out on the Illumina MiSeq platform, Nextera-XT, with paired-end 300 bp reads (Ramaciotti Centre, UNSW), to generate high coverage sequencing data for a more in-depth analysis of the diversity of viral sequences.

### Sequencing analysis

MiSeq pair-end reads were merged with PEAR (version 0.9.6) using default parameters (Zhang et al., 2014), non-overlapping reads and below a phred score of 30 discarded using Fastx version 0.0.14 (http://hannonlab.cshl.edu/fastx_toolkit/). Primer sequences (F: 26 nt, R: 20 nt) and sequences below 100 nt were removed. Sequences were collapsed at 100% nt identity and then used to generate insights in both the taxonomic affiliation and patterns of diversity of the ssRNA viruses associated with corals from the central GBR.

### Taxonomic affiliations and diversity–MCP

Taxonomic affiliations of virome reads were obtained using the Genome relative Abundance and Average Size GAAS Metagenomic tool (Angly et al., 2009) from MetaVir 2 (Refseq complete viral genomes protein sequences database from NCBI, release of 2015-01-05) (Roux et al., 2014). Taxonomic identity of reads to the MCP HcRNAV was confirmed by the additional BLASTn comparison against the RefSeq virus database from NCBI (bitscore < 50; *e*-values < 0.001).

Patterns of diversity rely on similarity cut-offs that cluster similar ssRNA virus sequences into operational taxonomic units (OTUs). In this study, patterns of diversity were estimated with two different approaches, each of them with a % similarity threshold. First, patterns of diversity were estimated with a 98% nucleotide similarity (default value Metavir2) cut-off for OTUs. Metavir is an online tool that does not allow modification of these parameters (that is why 98% was used instead of 97%). The resulting clustering distribution matrices from the Metavir2 pipeline were visualized in R (R Core Team, 2016) in a rarefaction scheme plot for the comparison of coral colonies and analyzed with a one-way PERMANOVA to evaluate OTU distribution. A high similarity threshold was selected to allow a visualization of rarefaction curves at the highest level of OTU diversity. If enough sequencing depth was obtained at high similarity threshold, then the same sequencing depth can be assumed from a lower diversity cut-off. Second, a complementary analysis used Quantitative Insights Into Microbial Ecology (QIIME Version 2.7.9) (Caporaso et al., 2010) and generated new OTUs with USEARCH (pick_otus.py) at a 65% similarity threshold. Although 97% nt similarity is the accepted cut-off for species level OTUs in bacterial communities based on the 16S rRNA marker gene (Vetrovsky and Baldrian, 2013), the recommended species-level cut-off is not known for the target sequence of the MCP HcRNAV. RNA viruses have fast rates of evolution (Holland et al., 1982; Duffy et al., 2008), therefore a conservative similarity cut-off of e.g., 65% may generate a glimpse into appropriate OTU diversity and community composition. OTU counts (i.e., relative abundance) were transformed into percentage values.

To further confirm taxonomic affiliations, a phylogenetic analysis was performed using the consensus nucleotide sequences from the main five largest OTUs (Supplementary Material: Table S1).

The sequences were aligned with ClustalW algorithm (Thompson et al., 1994) together with published data of relevant studies that obtained transcripts of ssRNA dinorna-like virus-like MCP reads from viromes of the coral *Montastraea cavernosa* and expressed sequence tag (EST) libraries from *Symbiodinium* cultures (SRA05206|GAIR4WKO3F1XL6) (Correa et al., 2013), an RNA viral metagenome from *A. tenuis* (gnl|SRA|SRR1210580.847558.2) (Weynberg et al., 2014), and cultured *Symbiodinium* RNAseq data (TR74740|c13_g1_i1) (Levin et al., 2016). The resulting alignment was used to construct a maximum likelihood tree (substitution model based on lowest Bayesian Information Criterion (BIC): kimura 2-parameter with gamma distributed rate variation), with 1,000 bootstrap replication steps using MEGA7 default parameters (Kumar et al., 2016).

### *Symbiodinium* genotyping

To examine a possible link between *Symbiodinium* identity and ssRNA virus communities, *Symbiodinium* diversity was assessed for *P. lutea* colonies via high-throughput sequencing of the internal transcribed spacer 2 (ITS2) region (Arif et al., 2014). Material for analysis was not available for the coral samples from Orpheus Island. Merged reads were clustered into OTUs at 97% sequence similarity (“cluster_fast” algorithm) using USEARCH Version 8.1.1812 (Edgar, 2010) with default parameters. Taxonomic affiliations of OTUs were acquired through a BLASTn search against the NCBI ‘nr' database. For all respective samples, the top three most abundant OTUs within every *Symbiodinium* sp. clade were aligned with ClustalW and analyzed for phylogenetic relation using a maximum likelihood tree with 1,000 bootstrap replication steps and a substitution model kimura 2-parameter with gamma distributed rate variation, based on lowest Bayesian Information Criterion (BIC).

## Results and discussion

### Taxonomic affiliations and diversity–MCP

Although SISPA-amplified DNA and the MCP amplicon were successfully synthesized for *A. tenuis, F. fungites, G. fascicularis, P. cylindrical*, and *P. damicornis* from Orpheus Island, PCR amplication failed for *A. hyacinthus, G. aspera* and *A. millepora*. Similarly, SISPA- amplified DNA and the corresponding ~500 bp amplicon were obtained from only six (A, B, C, D, G, H) of the eight colonies of *P. lutea*. Of the 354,795 unique sequences obtained from 11 coral colonies, 99.8% showed closest taxonomic affiliation with the ssRNA dinorna-like virus, HcRNAV (GenBank: LC120626.1) based on a BLASTx search to viral RefSeq NCBI (bitscore = 50 using MetaVir2 in December). The small number of other detected taxa (i.e., dsDNA viruses: *Iridoviridae, Phycodnaviridae*), likely reflects non-specific amplification due to the presence of several ambiguous positions in the primers used. These results confirm the high level of specificity of the PCR primers to dinorna-like virus and the efficacy of this PCR-based assay to assess presence and diversity of this viral group in corals.

**Figure 1:**
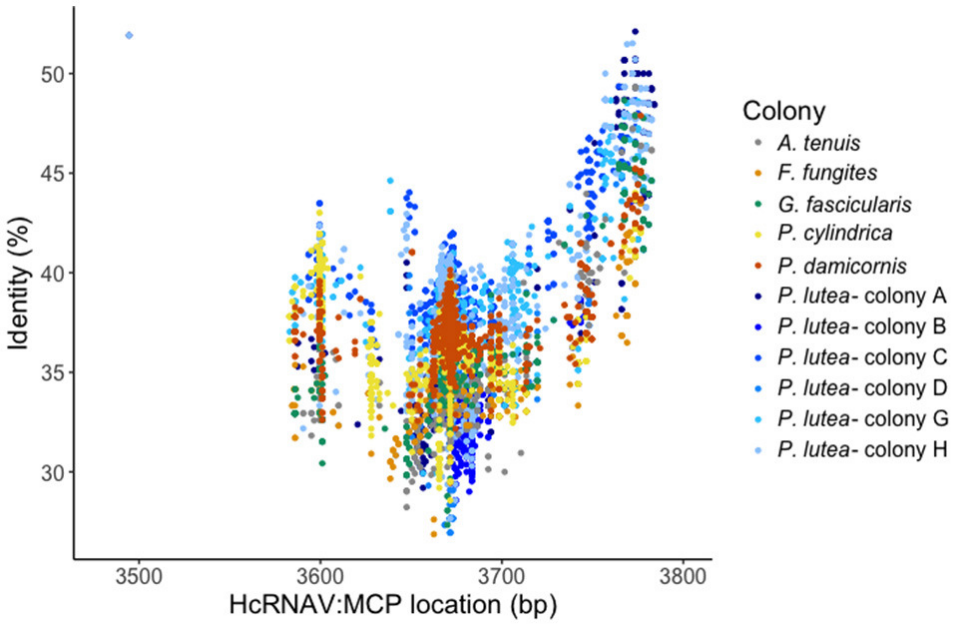
Major capsid protein (MCP) recruitment plot. Individual nt similarities from each read of each sample (colony) with its particular mean distribution along the MCP gene (X-axis), plotted against a % of identity to that specific region (Y-axis). Distribution displays in which region the amplified sequences are and how similar they are to the HcRNAV: MCP gene.

The level of sequence diversity we observed was high, with amino acid sequence similarities of 26–52% to the HcRNAV MCP gene (genome NC_007518; length = 4375 bp; type = linear) when comparing against a NCBI Refseq-virus database using the default recruitment plot algorithm from MetaVir2 (Figure 1). Sequences available through NCBI ref: (SRS2350274: SRX2999352, SRX2999353, SRX2999350, SRX2999355, SRX2999348, SRX2999349, SRX2999357, SRX2999351, SRX2999356, SRX2999358, SRX2999354). This scatter plot presents each read similar to the genome (MCP gene) as a dot, and displays the BLAST bitscore associated with the similarity detected.

**Figure 2:**
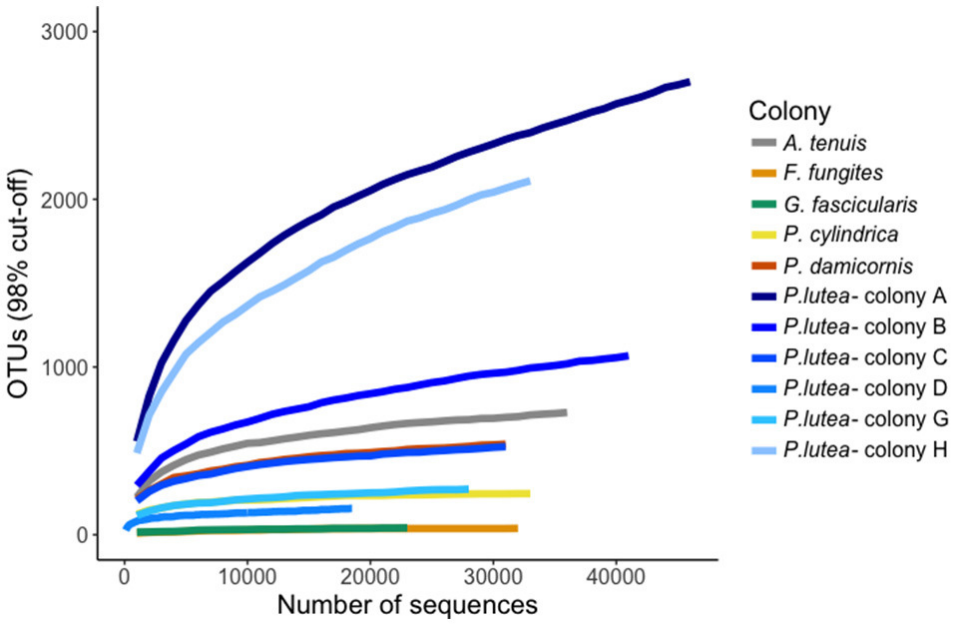
Diversity analysis of MiSeq MCP reads. Rarefaction plots were generated through MetaVir 2 to illustrate the variation of OTUs within the coral colonies analyzed. Using a similarity threshold of 98% within colonies of *P. lutea* (top) and among a colony of different corals (bottom).

Rarefaction plots using OTUs with 98% similarity cut-off showed the appropriateness of sequencing depth (i.e., plateau effect) for the majority of samples (Figure 2), which is important to avoid underestimation of similarities within and between samples (Sims et al., 2014). The exceptions were *P. lutea* colonies A and G from Davies Reef, and also *A. tenuis* and *P. damicornis* samples from Orpheus Island. A 98% threshold resulted in fewer than 1,000 OTUs in four of the six colonies of *P. lutea* and 2,000–3,000 OTUs for the remaining two colonies (A, G). The species, *F. fungites* and *G. fascicularis* had similar diversity levels of ~100 OTUs, while *P. cylindrica* showed a higher diversity with ~200 OTUs. A higher level of diversity was found for *P. damicornis* (~500 OTUs) and *A. tenuis* (~700 OTUs).

**Figure 3:**
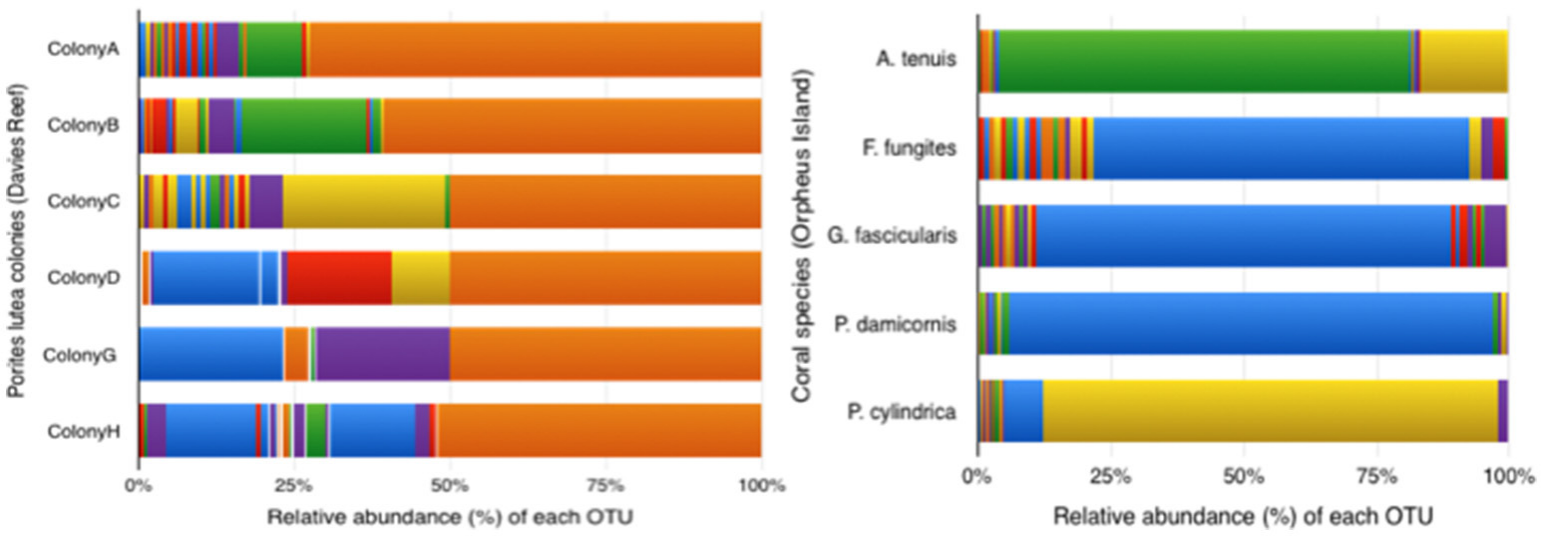
MCP sequence OTU composition using a 65% similarity cut-off. Total number of OTUs = 417. The same color in different samples represents the same OTU. **(Left)** *P. lutea* colonies from Davies Reef; **(Right)** colonies of different species from Orpheus Island.

The alternative OTU community composition based on a 65% clustering cut-off (total: 417 OTUs) revealed that the majority of samples have most of their MiSeq reads grouped into a small number of abundant OTUs (Figure 3), providing insight into the evenness (or lack thereof) of the ssRNA virus communities in the coral samples analyzed here. All of the *P. lutea* colonies from Davies Reef revealed a similar main OTU that included ~75% of the sequences, while the remaining sequences were clustered in several small OTUs.

The ssRNA virus community composition in the colonies used in this study was significantly different between locations based on the evaluation of score matrices with the Bray-Curtis index method (one-way PERMANOVA *F* = 0.061; df = 1; *p* = 0.0044). Colonies from Orpheus Island showed an OTU distribution represented by one large OTU (~75% of reads) with the remaining 25% of reads being distributed across several small OTUs. Sequences from *F. fungites, G. fascicularis*, and *P. damicornis* revealed a similar relative abundance of the same main OTU (the blue OTU in Figure 3), suggesting they may harbor similar *Symbiodinium* communities; *Symbiodinium* types C1 and C3 have been reported as the dominant *Symbiodinium* types for these corals (Tonk et al., 2013). *Acropora tenuis* and *P. cylindrica* harbored a different main OTU (however, the literature suggests C3 as the dominant *Symbiodinium* clade for *A. tenuis* and C15 and C1 for *P. cylindrica*) (Tonk et al., 2013). The virus community composition was significantly different among these five colonies (Figure 3; one-way PERMANOVA *F* = 0.036; *df* = 5; *p* = 0.0202).

**Figure 4:**
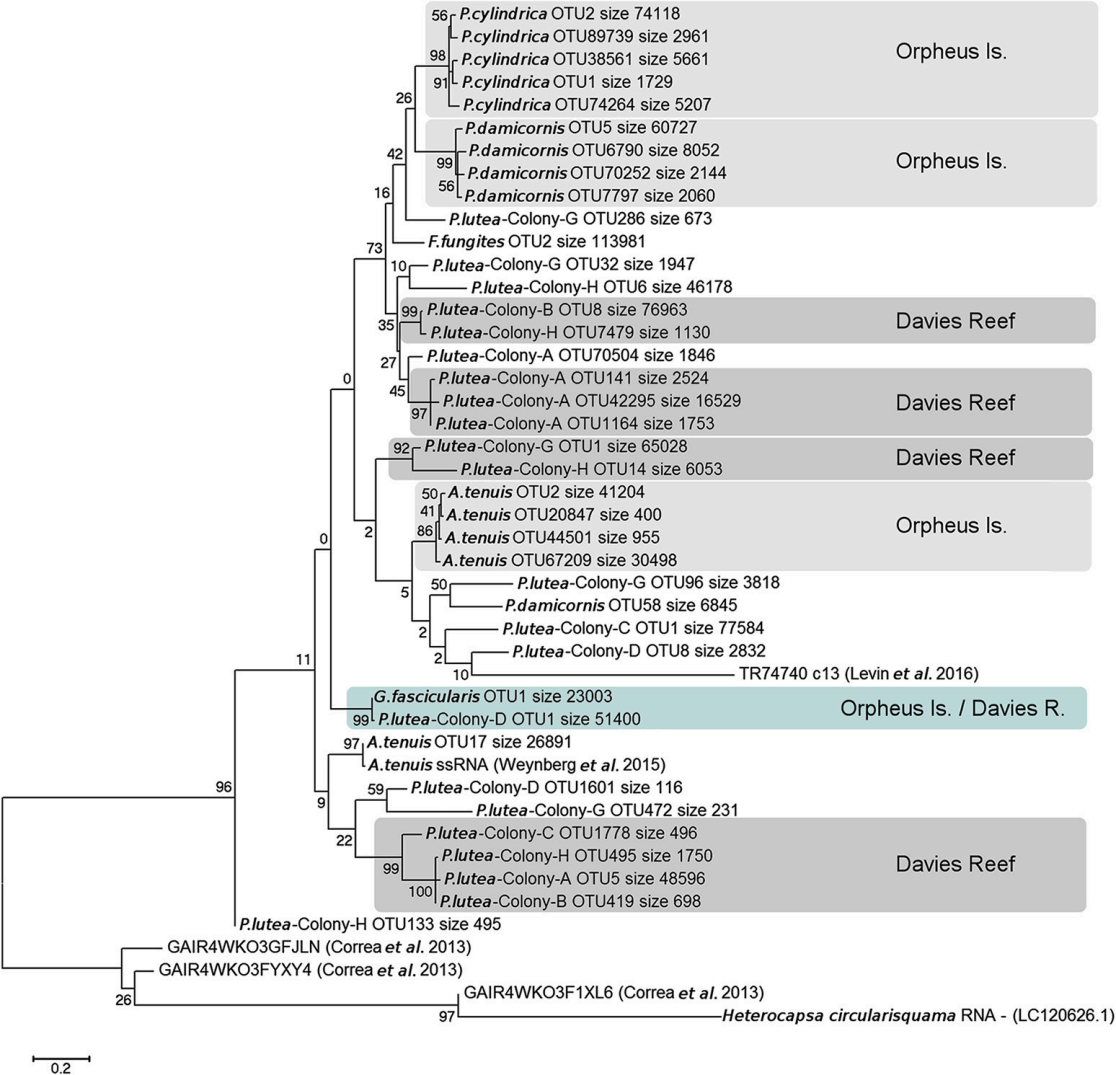
Unrooted maximum likelihood tree displaying major capsid protein phylogenetics. Phylogenetic relationships are shown for major OTUs based on dinorna-like virus MCP sequences obtained in this study and from publicly available relevant coral and *Symbiodinium* viromes. Bootstrap values are shown next to the branches and ‘size' represents the number of sequences found for each OTU. Highlighted are OTU clusters with well-supported bootstrap values (>85%) with their sampling location; light-gray for Orpheus Is, dark-gray for Davies Reef and light-blue for viral communities that were found in hosts from both locations.

The phylogenetic analysis of sequences from the most representative OTUs at 97% similarity cut-off, revealed a strong pattern of congruence between viral relatedness and coral host taxonomy (Figure 4), suggesting coral host taxonomy reflects *Symbiodinium* identity as the virus targets *Symbiodinium* and not the coral. This species-specific clustering pattern was observed for the majority of the most representative OTUs (i.e., relative abundance of sequences per OTU over the total number of OTUs). Despite this, the multi-colony analysis of *P. lutea* illustrated how different OTU composition can be among also conspecific colonies (e.g., colonies A, B, C, and H). In some cases, all OTUs obtained from a coral species fell within a single lineage (e.g., *F. fungites*), and additional OTUs were found to deviate from the coral host species-specific pattern. This analysis takes into account only the largest OTUs (i.e., the top five most representative OTUs) under a restrictive and more specific 97% cut-off, therefore allowing a comparison of OTU distribution among linages (Supplementary Material: Table S1). The bootstrap values on the basal nodes in the phylogenetic tree were below 80%, therefore, caution should be taken in drawing any conclusions on relatedness among terminal clades. The comparison of our data with publicly available dinorna-like virus MCP sequences from coral and *Symbiodinium*, showed these were generally more distantly related, which is unsurprising given these came from other regions in the world. The exceptions were the sequences from Weynberg et al. (2014) and Levin et al. (2016). The former are derived from the same *A. tenuis* samples used in our study and further confirm the success and specificity of our PCR assay, while the latter were obtained from a *Symbiodinium* C1 culture obtained from an *A. tenuis* colony collected from an inshore reef in the central GBR. The lack of colony replication for the coral species from Orpheus Island prevents a comparative analysis between locations. However, OTUs from the corresponding samples were allocated into separated clusters based on their collection location (i.e., supported by high bootstrap values) and no overlap was found for the majority of comparisons. Interestingly, OTUs obtained from *G. fascicularis* (Orpheus Island) and *P. lutea* colony D (Davies Reef) clustered with a 99% bootstrap value, which provides insights into the presence of similar viral communities present in different species and locations.

*Symbiodinium* type C15 is the most common endosymbiont of *P. lutea* and other Poritidae (e.g., *P. cylindrical*; http://www.SymbioGBR.org, Tonk et al., 2013). Our ITS2 sequence analysis demonstrates that all *P. lutea* colonies examined here were dominated by *Symbiodinium* C15, suggesting that the abundance of a single, dominant ssRNA virus OTU matches the *Symbiodinium* host diversity (Supplementary Material: Table S2). The presence/absence of background types (i.e., relative abundance < 1%) did not have an influence on the dinorna-like virus diversity. In addition, since all *Porites* colonies harbored the same dominant *Symbiodinium* type (Supplementary Material: Table S2, Figure S1), the failure of PCR amplication in *Porites* colonies E and F indicates that not all *Symbiodinium* communities harbored by the corals we sampled were infected with this virus. The latter notion is supported by the observation that dinorna-like virus MCP transcript was among the most highly expressed genes in a *Symbiodinium* C1 population isolated from the coral, *A. tenuis*, from the Whitsundays, while it was only just detectable in the transcriptome of another *Symbiodinium* C1 population from Magnetic Island (Levin et al., 2016). Further studies of corals associated with a wider diversity of *Symbiodinium* types are required to confirm these findings.

### Primer specificity

Unfortunately no control samples from seawater and other organisms were included to test the specificity of the primers to viruses of the coral holobiont (i.e., the coral and all of its associated symbionts). However, the primers were designed specifically to viruses isolated from corals and amplified sequences that were divergent from HcRNAV sequences in public databases. While it is likely that the primers are specific to viruses that reside in the coral holobiont and that target *Symbiodinium*, it is possible that these primers work on other organisms that live in symbiosis with *Symbiodinium*, such as clams, Foraminifera and sponges. Further studies are required to determine the specificity of these primers.

## Biological implications and conclusions

Previous genomic evidence indicates that ssRNA viruses are part of the viral assemblages associated with corals and their dinoflagellate endosymbionts (Correa et al., 2013; Weynberg et al., 2014; Wood-Charlson et al., 2015; Levin et al., 2016). Our results confirm that ssRNA viruses with dinorna-like MCP genes are commonly associated with corals on the GBR, and suggest that some level of location- and host-specificity exists.

Early coral virus studies have shown that virus consortia are highly diverse at the whole community level (Angly et al., 2006; Marhaver et al., 2008), but no previous studies have examined the level of diversity of populations of a single virus that is associated with corals and targets the endosymbiotic *Symbiodinium*. Our results show such populations can be highly diverse, as indicated by the many OTUs identified here within colonies. Although, the methodology present in this study unveils the diversity of a particular type of ssRNA virus in corals, it does not allow its abundance to be assessed. We recommend that further studies focus on the development of digital or quantitative PCR to evaluate absolute virus abundance.

The main rationale behind this research was the hypothesis that algal viruses may play a role in coral health by targeting the algal endosymbionts and may therefore be linked to coral bleaching events (Sutherland et al., 2004; Thurber and Correa, 2011). Neighboring conspecific coral colonies exposed to the same conditions can differ markedly in their bleaching responses (Edmunds, 1994). This patchy spatial distribution can be caused by different *Symbiodinium* communities hosted by the coral (Blackall et al., 2015), but may also reflect the fact that not all colonies are infected by the ssRNA virus targeting *Symbiodinium*. The novel and relatively easy-to-use assay presented here, which can be further developed into a quantitative PCR assay to assess abundance in space and time, will facilitate an examination of the hypothesis that coral bleaching occurs in response to a combination of environmental stressors and viral infection of *Symbiodinium*.

## Author contributions

JM conducted the experiments and laboratory analysis. JM and PB performed the bioinformatic analysis. JM wrote the first draft and all authors contributed substantially to revisions and interpretation of data of the work. MJHvO and KDW conceived the study.

### Conflict of interest statement

The authors declare that the research was conducted in the absence of any commercial or financial relationships that could be construed as a potential conflict of interest.
