# Supplementary material for: A PCR-Based Assay Targeting the Major Capsid Protein Gene of a Dinorna-Like ssRNA Virus That Infects Coral Photosymbionts
Source: Front Microbiol. 2017 Sep 1;8:1665. doi: 10.3389/fmicb.2017.01665 (PMC5585145; doi:10.3389/fmicb.2017.01665)

Supplementary material

Table S1 Dinornavirus major capsid protein operational taxon units (OTU). Sequences were clustered at a 97% sequence similarity and analysed with a maximum likelihood phylogenetic tree. The link in the table leads to the untrimmed OTU sequences with references in FASTA file.

OTU Fasta files for the corresponding samples	https://figshare.com/s/bd99f0a5da0fb1331dc2

Table S2 Relative abundance of *Symbiodinium* in *Porites* colonies. Clade C was the dominant *Symbiodinium* type in all sampled *Porites* colonies. Several background clades were detected with low relative abundance.

Symbiodinium	***Porites* colony [%]**							
	**A**	**B**	**C**	**D**	**E**	**F**	**G**	**H**
Clade A	0.000	0.002	0.000	0.000	0.007	0.001	0.000	0.001
Clade B	0.326	0.047	0.000	0.000	0.009	0.022	0.000	0.004
Clade C	99.673	99.951	100.000	99.999	99.980	99.977	100.000	99.995
Clade D	0.001	0.000	0.000	0.000	0.001	0.000	0.000	0.000
Clade G	0.000	0.000	0.000	0.001	0.003	0.000	0.000	0.000

Figure S1. Phylogenetic relation of *Symbiodinium* sp. clades in *Porites* colonies. Clade C cluster the majority of OTUs in all sampled *Porites* colonies. Method: Maximum likelihood tree with 1000 bootstrap replication steps and a substitution model kimura 2-parameter with gamma distributed rate variation, based on lowest Bayesian Information Criterion (BIC).

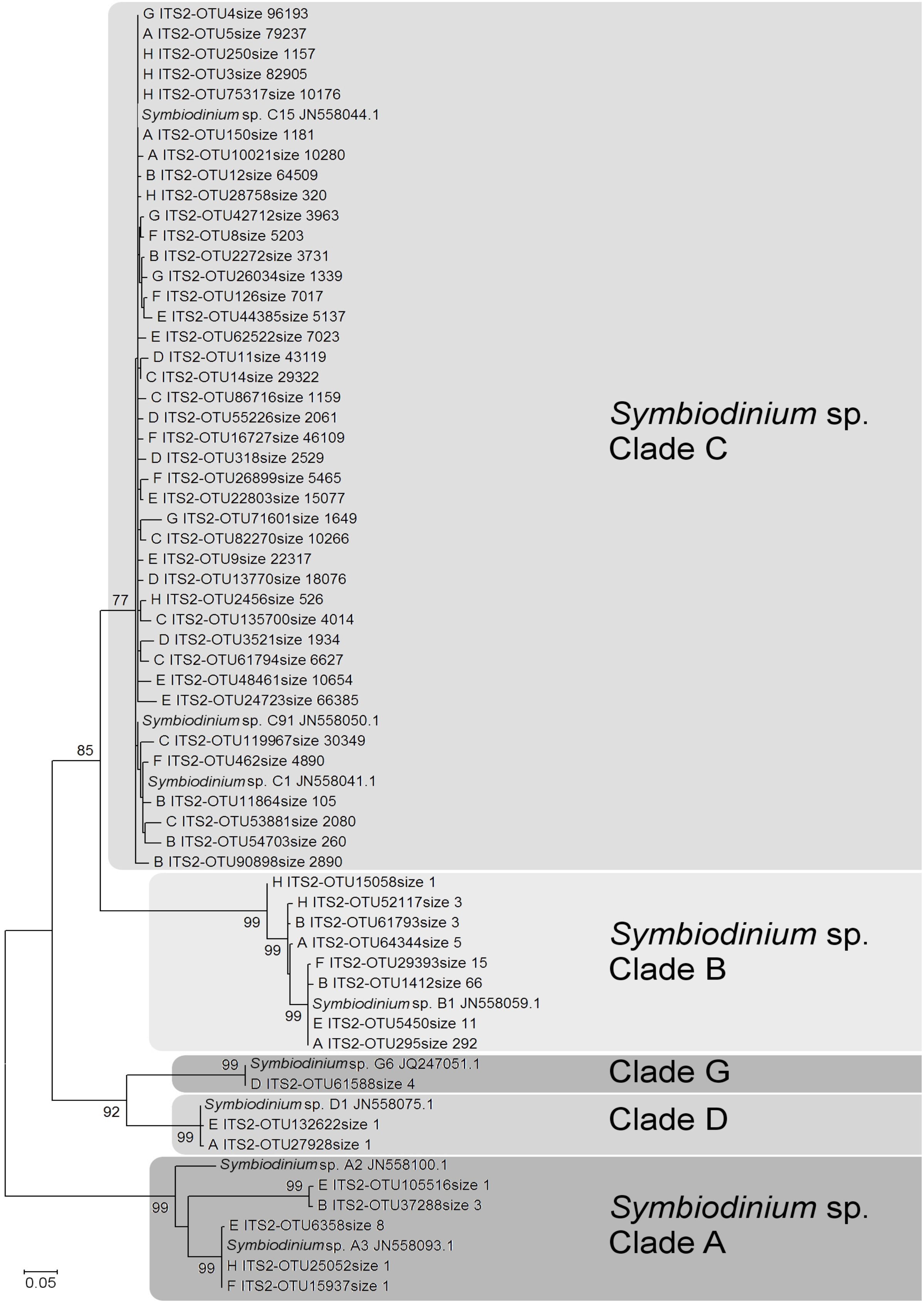

Supplement: Supplementary file 1 [file Table1.pdf]
